# Population pharmacokinetics of amodiaquine and piperaquine in African pregnant women with uncomplicated *Plasmodium falciparum* infections

**DOI:** 10.1002/psp4.13211

**Published:** 2024-09-03

**Authors:** Junjie Ding, Richard M. Hoglund, Harry Tagbor, Halidou Tinto, Innocent Valéa, Victor Mwapasa, Linda Kalilani‐Phiri, Jean‐Pierre Van Geertruyden, Michael Nambozi, Modest Mulenga, Sebastian Hachizovu, Raffaella Ravinetto, Umberto D'Alessandro, Joel Tarning

**Affiliations:** ^1^ Mahidol Oxford Tropical Medicine Research Unit, Faculty of Tropical Medicine Mahidol University Bangkok Thailand; ^2^ Centre for Tropical Medicine and Global Health, Nuffield Department of Clinical Medicine University of Oxford Oxford UK; ^3^ University of Health and Allied Sciences Ho Ghana; ^4^ Clinical Trial Unit Nanoro Nanoro Burkina Faso; ^5^ Department of Community and Environmental Health, Kamuzu University of Health Sciences Blantyre Malawi; ^6^ Global Health Institute University of Antwerp Antwerp Belgium; ^7^ The Tropical Diseases Research Center Ndola Zambia; ^8^ Public Health Department Institute of Tropical Medicine Antwerp Belgium; ^9^ School of Public Health University of the Western Cape Cape Town South Africa; ^10^ MRC Unit The Gambia at the London School of Hygiene and Tropical Medicine Fajara Gambia; ^11^ The WorldWide Antimalarial Resistance Network Oxford UK

## Abstract

Artemisinin‐based combination therapy (ACT) is the first‐line recommended treatment for uncomplicated malaria. Pharmacokinetic (PK) properties in pregnant women are often based on small studies and need to be confirmed and validated in larger pregnant patient populations. This study aimed to evaluate the PK properties of amodiaquine and its active metabolite, desethylamodiaquine, and piperaquine in women in their second and third trimester of pregnancy with uncomplicated *P. falciparum* infections. Eligible pregnant women received either artesunate‐amodiaquine (200/540 mg daily, *n* = 771) or dihydroartemisinin‐piperaquine (40/960 mg daily, *n* = 755) for 3 days (NCT00852423). Population PK properties were evaluated using nonlinear mixed‐effects modeling, and effect of gestational age and trimester was evaluated as covariates. 1071 amodiaquine and 1087 desethylamodiaquine plasma concentrations, and 976 piperaquine plasma concentrations, were included in the population PK analysis. Amodiaquine concentrations were described accurately with a one‐compartment disposition model followed by a two‐compartment disposition model of desethylamodiaquine. The relative bioavailability of amodiaquine increased with gestational age (1.25% per week). The predicted exposure to desethylamodiaquine was 2.8%–32.2% higher in pregnant women than that reported in non‐pregnant women, while day 7 concentrations were comparable. Piperaquine concentrations were adequately described by a three‐compartment disposition model. Neither gestational age nor trimester had significant impact on the PK of piperaquine. The predicted exposure and day 7 concentrations of piperaquine were similar to that reported in non‐pregnant women. In conclusion, the exposure to desethylamodiaquine and piperaquine was similar to that in non‐pregnant women. Dose adjustment is not warranted for women in their second and their trimester of pregnancy.

**Study Highlights**

**WHAT IS THE CURRENT KNOWLEDGE ON THE TOPIC?**

Artemisinin‐based combination therapy (ACT) is the first‐line recommended treatment for uncomplicated malaria. Only few clinical trials have assessed the pharmacokinetic properties of antimalarial drugs in pregnant patients.

**WHAT QUESTION DID THIS STUDY ADDRESS?**

This study was a phase 3 clinical trial to study the efficacy and safety of ACTs in pregnant women with malaria. Population pharmacokinetics of two antimalarial drugs, amodiaquine and piperaquine, was evaluated with nonlinear mixed‐effect modeling to assess the impact of gestational age.

**WHAT DOES THIS STUDY ADD TO OUR KNOWLEDGE?**

The analysis indicated that the relative bioavailability of amodiaquine increased with gestational age, while there was no change in the pharmacokinetics of piperaquine. However, drug exposures were slightly higher than that reported in non‐pregnant women. The study confirmed that pregnancy had no clinically significant impact on the exposure to piperaquine and amodiaquine.

**HOW MIGHT THIS CHANGE DRUG DISCOVERY, DEVELOPMENT, AND/OR THERAPEUTICS?**

Based on these findings, the current standard dosing regimen of amodiaquine and piperaquine should be effective in pregnant women with uncomplicated malaria, and dose adjustment is not warranted.

## INTRODUCTION

An estimated 249 million malaria cases and 608,000 malaria‐related deaths were reported in 2022.^1^ Approximately 96% of the globally estimated cases are due to *Plasmodium falciparum (P. falciparum)*, and the majority of the disease burden occur in sub‐Saharan Africa.^1^ Pregnant women are more susceptible to malaria than the general population,^2^, ^3^ largely attributed to suppressed immunity during pregnancy and the presence of a malaria‐naïve organ resulting in a high degree of sequestration in the micro‐vasculature in the placenta.^3^ The estimated odds ratio (OR) of *P. falciparum* infections in pregnant women compared to non‐pregnant women has been reported to range between 2.11 and 2.34.^3^ Malaria infection during pregnancy is a significant public health problem with an estimated 35 million pregnant women being at risk for malaria.^4^ Malaria during pregnancy is also highly correlated with adverse risks for the fetus, resulting in low birth weight, increased risk of stillbirth, developmental consequences and increased risk of infant mortality.^4^, ^5^

A standard three‐day course of artemisinin‐based combination therapies (ACTs) is the current first‐line treatment for pregnant women in their second and third trimester with uncomplicated *P. falciparum* malaria.^6^ The artemisinin component kills the majority of parasites during the 3 days of treatment to quickly resolve clinical symptoms and the longer‐acting partner drug (i.e., lumefantrine, piperaquine, amodiaquine, or mefloquine) eliminates the residual parasites to prevent recurrent infections. Sub‐optimal dosing could result in low drug levels, which is a major driver of treatment failures and the development of drug resistant parasites.

Pregnancy is linked to significant physiological changes. Indeed, many studies indicate that pregnancy is associated with prolonged gastric emptying and gastrointestinal transit time, increased cardiac output, elevated blood volume, increased volume of body fluid spaces, decreased plasma albumin concentration, altered hormone levels, and increased renal blood flow.^7^ Expression and/or abundancy of drug metabolizing enzymes including phase I or phase II enzymes could change during pregnancy.^7^ These physiological and enzymatic changes might have notable impact on the ADME process of drug in the body, as a result, affecting clinical outcome. It is crucial to assess pregnant‐relevant PK changes for partner drugs in malaria therapy to avoid sub‐optimal or toxic exposure. However, only a few studies have investigated the PK characteristics of amodiaquine,^8^, ^9^ piperaquine,^10^, ^11^, ^12^, ^13^ mefloquine,^14^, ^15^ and lumefantrine^16^, ^17^, ^18^, ^19^, ^20^, ^21^, ^22^ in pregnant women, and the trials are often small. Thus, these results need to be confirmed and validated in a large pregnant patient population in phase 3 trials.

The PREGACT Study (ClinicalTrials.gov number: NCT00852423) was a large phase 3 clinical efficacy and safety trial, conducted in 3428 pregnant women infected with acute uncomplicated *P. falciparum* malaria.^23^ Along with the assessment of the treatment outcome of 4 ACTs (artemether‐lumefantrine, dihydroartemisinin‐piperaquine, artesunate‐amodiaquine, and artesunate‐mefloquine), PK sampling of the 4 partner drugs were collected, allowing to characterize the PK properties in malaria‐infected women in their second and third trimester pregnancy. The aim of the current sub‐study was to investigate the effect of gestational age on the PK properties of the two antimalarial partner drugs amodiaquine and piperaquine in African pregnant women with malaria, and if needed, to optimize dose regimens.

## METHODS

### Study design

This was a non‐inferiority, multi‐center, randomized, open‐label clinical trial to assess efficacy and safety of 4 ACTs in pregnant women in Africa with malaria. The trial was conducted between June 2010 and August 2013 in four sub‐Saharan African countries: Burkina Faso (two sites), Ghana (three sites), Malawi (one site), and Zambia (one site). Briefly, pregnant women in the second or third trimester and with a *P. falciparum* mono‐infection were eligible and randomly assigned to one of the following four treatments: artemether–lumefantrine, artesunate–amodiaquine, artesunate–mefloquine, or dihydroartemisinin–piperaquine (870 patients for each treatment). The trial was approved by the ethics committee at the Antwerp University Hospital, the relevant national or local ethics committees, and the national drug regulatory authorities. The details of the study (such as patient eligibility and clinical assessment) have been published already.^23^ This article presents the pharmacokinetic results of amodiaquine and piperaquine.

### Drug regimen

The eligible pregnant women were given the antimalarial drugs under direct observation for 3 consecutive days. Women enrolled in artesunate‐amodiaquine group received a daily dose of 2 tablets of artesunate‐amodiaquine (Sanofi‐Aventis, France). One tablet contains 100 mg artesunate and 352.64 mg amodiaquine salt (270 mg of amodiaquine base). Women in the dihydroartemisinin‐piperaquine group received a daily dose of 3 tablets (Sigma‐Tau, Industrie Farmaceutiche Riunite S.p.A, Italy). One tablet contains 40 mg of dihydroartemisinin and 320 mg of piperaquine tetraphosphate (171 mg piperaquine base). A dose was repeated in full if vomiting occurred within 30 min of administration and halved if vomiting occurred between 30 min and 1 h post‐dosing. Patients who vomited at any dose were excluded from the PK modeling analysis.

### Blood samples

A venous blood sample (2 mL) was collected from all women on day 7, and additional pharmacokinetic samples were collected at other clinical visits when possible. Blood samples were centrifuged at 2000 ***g***, and the plasma was stored at −20°C. The samples were transferred within 2 months to a −80°C freezer and thereafter shipped to the department of clinical pharmacology at Mahidol Oxford Tropical Medicine Research Unit, Bangkok, Thailand, for analyses.

## Concentration quantification

The plasma concentrations of amodiaquine/desethylamodiaquine and piperaquine were measured using two validated LC/MS–MS methods. The details of assays for amodiaquine/desethylamodiaquine^24^, ^25^ and piperaquine^26^ have previously been published. Three levels of internal quality control samples were analyzed in triplicates with each batch (96‐well plate) of clinical samples to ensure accurate and precise drug measurements of clinical trial samples.

### Population PK analysis

Observed drug concentrations were transformed into their natural logarithms for the analysis. The population PK analysis was performed using NONMEM (version 7.4, ICON Development Solutions, Ellicott City, MD, USA), which was compiled using gFortran (version 4.60). R (version 3.2.0, http://www.r‐project.org/) was used for visualizations and model evaluations. The first‐order conditional estimation method including η‐ε interaction (FOCE‐I) was used throughout the model‐building procedure. Perl‐speaks NONMEM (PsN; version 4.6.0), and Pirana (Version 2.9.3) were used to evaluate the goodness of fit during the model‐building process.

If the fraction of PK samples with concentrations below the lower limit of quantification (LLOQ) was low (i.e., <5% of total samples), these LLOQ samples were omitted directly from the model development process. Otherwise, different methods were investigated to handle the censored data as proposed by Beal,^27^ including M1 (excluded from model), M6 (imputing the first concentration below the LLOQ with half of the LLOQ, then discarded the remaining LLOQ data) and M3 (maximizing the likelihood of predicting censored data to be below the LLOQ). Categorical visual predictive checks were used to compare the predicted and observed data below the LLOQ.

First‐order elimination from the central compartments was assumed for all drugs modeled. Amodiaquine and its active metabolite, desethylamodiaquine, were modeled with a joint parent‐metabolite model, assuming complete bioconversion of amodiaquine into desethylamodiaquine. All possible combinations of one‐, two‐, three‐ compartment models were investigated. Due to uninformative sample collection in absorption phase, absorption relevant parameters such as the absorption rate (ka), mean transit time (MTT), and number of transit compartments were fixed to literature values for both amodiaquine^9^ and piperaquine.^28^ Additionally, for piperaquine, the effect of dose occasion on the relative bioavailability was fixed to 23.7% increase with each dose, as reported in a large individual‐level data population pharmacokinetic meta‐analysis.

Inter‐individual variability (IIV) was modeled exponentially for all PK parameters (Equation 1)

(1) $$\theta_{i}=\theta \times \exp\left(\eta_{i,\theta }\right)$$

where *θ*_*i*_ is the individual parameter estimate for the *i*th individual, *θ* is the population parameter estimate, *η*_*i,θ*_ is the IIV in parameter *θ* for individual *i*, individual *η*_*θ*_ is assumed to be normally distributed with a zero mean and variance *ω^2^*. Relative bioavailability (F) was fixed to unity (100%) for the population to allow the estimation of IIV of the absorption. Residual unexplained variability was modeled with an additive error on the log‐transformed concentrations, which is essentially equivalent to an exponential residual error on an arithmetic scale.

### Covariates model

The covariates of interest included bodyweight, gestational age (GA, estimated by the fundal height), age and parasitemia at recruitment.

First, body weight was added on all clearance and volume parameters using an allometric function with a fixed exponent of 0.75 and 1, respectively (Equations 2 and 3).

(2) $$\theta_{i}=\theta \times \exp\left(\eta_{i,\theta }\right)\times {\left({\mathrm{BW}}_{i}/{\mathrm{BW}}_{\text{median}}\right)}^{0.75}$$

(3) $$\theta_{i}=\theta \times \exp\left(\eta_{i,\theta }\right)\times \left({\mathrm{BW}}_{i}/{\mathrm{BW}}_{\text{median}}\right)$$

where BW_*i*_ is individual body weight and BW median is the median body weight of the population.

Then, gestational age, a continuous covariate, was evaluated on all pharmacokinetic parameters using a linear function, spline and power function. Finally, other covariates (age, baseline parasite density) were evaluated on all PK parameters. All covariates, except the allometric function of body weight, were analyzed in a stepwise manner with a forward selection step (*p* = 0.01, *df* = 1, ∆OFV = 6.63) and a stricter backward elimination step (*p* = 0.001, *df* = 1, ∆OFV = 10.83).

### Model evaluation

Basic goodness‐of‐fit diagnostics were used to evaluate potential systematic errors and model misspecifications. The predictive performance of final models was evaluated using simulation‐based diagnostics (i.e., visual predictive checks, *n* = 2000). Additionally, the uncertainty of the final population PK models was assessed by a bootstrap approach (*n* = 1000), and descriptive statistics (median and the 2.5th–97.5th percentiles) were calculated and compared with the values obtained in NONMEM.

### In silico simulation

Since non‐pregnant women were not enrolled in this phase 3 study, head‐to‐head comparison of exposures between non‐pregnant and pregnant women was not possible. In order to address this question, we simulated an expected exposure in non‐pregnant women based on PK parameters in 2 published pooled population PK meta‐analyses for amodiaquine^29^ and piperaquine.^28^ The results of these simulations were then compared with the exposures in pregnant women based on the developed models. Desethylamodiaquine exposure was used after amodiaquine treatment as this active metabolite has a substantially higher exposure, compared to amodiaquine, and is responsible for the therapeutic efficacy.

## RESULTS

A total of 784 pregnant women with *P*. *falciparum* infection were included in the artesunate‐amodiaquine group, and 763 in the dihydroartemisinin‐piperaquine group. Thirteen (1.7%) and eight (1.0%) women who vomited after dosing in the artesunate‐amodiaquine and dihydroartemisinin‐piperaquine groups, respectively, were excluded from the PK modeling analysis. Demographic data are shown in Table 1 and were overall comparable between the two groups.

**TABLE 1:** Patient demographics in the clinical study of amodiaquine and piperaquine in pregnant women.

Characteristics	Amodiaquine	Piperaquine
Number of patients	771	755
Daily dose (mg/kg)	12.8 (6.8–19.1)	17.8 (8.3–27.4)
Age (years)	22 (15–43)	20 (15–43)
Bodyweight (kg)	55 (37–104)	54 (35–115)
Height (cm)	158 (132–179)	155 (138–178)
Gestational age (weeks)	24 (13–36)	24 (16–36)
Trimester 2 [*n* (%)]	581 (75.7)	519 (69.1)
Parasitemia at enrollment (parasites/μL)	560 (0–82,292)	680 (5–355,400)
Gametocytes at enrollment (parasites/μL)	0 (0–253)	0 (0–1200)

*Note*: Data are reported as median (min–max), unless otherwise stated.

### PK of amodiaquine and desethylamodiaquine

A total of 1071 amodiaquine and 1087 desethylamodiaquine plasma samples were collected and measured for drug quantification. Of these, 848 (79.2%) and 29 (2.7%) measurements were below the LLOQ for amodiaquine and desethylamodiaquine, respectively. Amodiaquine and desethylamodiaquine plasma concentration‐time data were best described by one‐ and two‐compartment kinetics, respectively (Figure S1). The categorical visual predictive check showed a good prediction of data below the LLOQ when using the M1 method, for both amodiaquine and desethylamodiaquine. Samples below the LLOQ were therefore omitted during further model development.

Body weight was added a priori as a fixed allometric function on all clearance and volume of distribution parameters. Although it led to a slightly worse model fit, it was retained in the model as pregnant and non‐pregnant women are expected to have a systematically different body weight (∆OFV = 35.2). Inclusion of GA on F using a linear function was statistically significant (∆OFV = −15.6, *p* < 0.001), with 1.28% increase in F per week increase in GA. Using a power function or a spline function to describe the impact of GA on F did not improve the model fit further (*p* > 0.05). Considering pregnant women with 24 weeks of GA as a reference population, F was 90% (90%CI, 54%–148%) and 115% (90%CI, 70%–191%) for women at 16 and 36 weeks of GA, respectively.

Age and baseline parasite density showed no significant impact on the pharmacokinetics of either amodiaquine or desethylamodiaquine.

### Pharmacokinetics of piperaquine

A total of 982 piperaquine plasma samples were collected and measured for drug quantification, with 6 (0.6%) measurements below the LLOQ. These LLOQ samples were omitted directly from the population PK analysis. Piperaquine plasma concentrations were adequately described by a three‐compartment disposition model (Figure S2).

Body weight was added a priori as an allometric function on all clearance and volume of distribution parameters. Although it led to a slightly worse model fit, it was retained in the model as pregnant and non‐pregnant women are expected to have a systematically different body weight (∆OFV = 41.4). GA or trimester status were not significant covariates on any PK parameters. Baseline parasite density was identified as a significant covariate on F, with 11.6% decrease in F per one log unit increase of parasite density (∆OFV = −20.7, *p* < 0.001). Age had no impact on any parameter.

### Model evaluation

**FIGURE 1:**
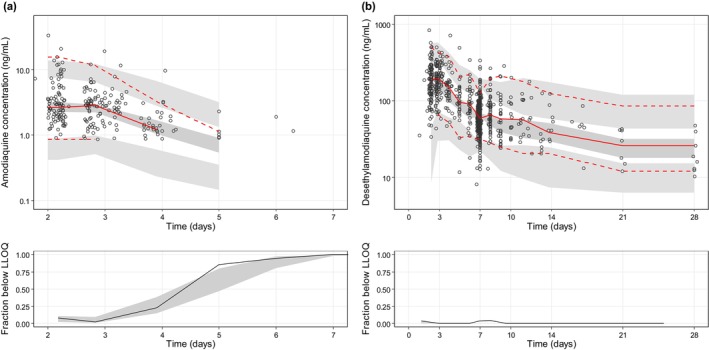
Visual predictive check of the final population pharmacokinetic model for amodiaquine (a) and desethylamodiaquine (b) based on 2000 stochastic simulations. Upper panel: Open circles represent the observations, and solid lines represent the 5th, 50th, and 95th percentiles of the observed data. The shaded areas represent the 95% confidence intervals around the simulated 5th, 50th, and 95th percentiles. Lower panel: Open circles represent observed proportion of censored data. The shaded areas represent the 95% predicted interval of censored data.

**FIGURE 2:**
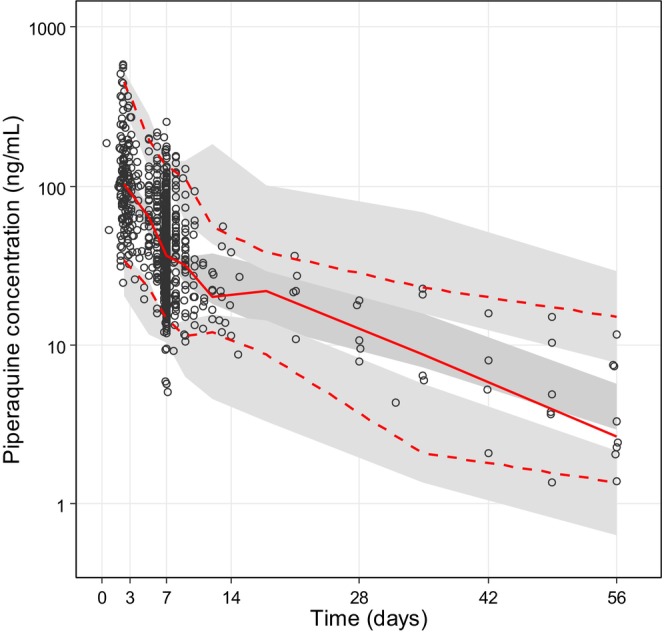
Visual predictive check of the final population pharmacokinetic model for piperaquine based on 2000 stochastic simulations. Open circles represent the observations, and solid lines represent the 5th, 50th, and 95th percentiles of the observed data. The shaded areas represent the 95% confidence intervals around the simulated 5th, 50th, and 95th percentiles.

Parameter estimates for amodiaquine/desethylamodiaquine and piperaquine had good precision, with small relative standard errors (Tables 2 and 3). Predicted secondary PK parameters (i.e., elimination half‐life and total exposure) derived from the final model are presented in Tables 2 and 3. Goodness‐of‐fit diagnostics showed no major discrepancy between observation and model prediction and no signal of model misspecification (Figures S3 and S4). Visual predictive checks (*n* = 2000) demonstrated that the final models captured adequately the central tendency and variability of the observations for both amodiaquine/desethylamodiaquine and piperaquine (Figures 1 and 2). Prediction‐corrected visual predictive checks (*n* = 2000) showed that the proposed model had an overall good predictive performance across the studied range of gestational ages for amodiaquine/desethylamodiaquine (Figure S5) and piperaquine (Figure S6).

**TABLE 2:** Final parameter estimates of amodiaquine and desethylamodiaquine population pharmacokinetics in pregnant women.

Parameter	NONMEM Estimates (%RSE^a^)	Bootstrap median (95%CI)	CV for IIV (%RSE^a^)	Bootstrap median (95%CI)	Shrinkage (%)
*Amodiaquine (AQ)*					
F_AQ_ (%)	100 fixed	–	30.6 (8.4)	30.4 (24.9–35.4)	33.7
Ka (1/h)	0.589 fixed	–	–	–	–
MTT (h)	0.236 fixed	–	–	–	–
Number of transit compartment	2 fixed	–	–	–	–
CL/F_AQ_ (L/h)	6780 (4.9)	6770 (6130–7440)	–	–	–
V_C_/F_AQ_ (L)	272,000 (8.9)	270,000 (228,000–322,000)	–		–
RUV	0.267 (13.9)	0.262 (0.200–0.338)	–	–	4.6
*Desethylamodiaquine (DEAQ)*					
CL/F_DEAQ_ (L/h)	38.3 (9.6)	38.0 (30.4–43.6)	19.7 (37.0)	19.2 (6.2–36.2)	70.4
V_C_/F_DEAQ_ (L)	861 (15.7)	897 (656–1240)	205 (13.4)	191 (103–318)	74.3
Q/F_DEAQ_ (L/h)	81.7 (7.0)	82.2 (72.1–94.5)	–	–	–
V_p_/F_DEAQ_ (L)	13,200 (13.4)	13,400 (11,200–17,100)	–	–	–
RUV	0.122 (10.7)	0.122 (0.096–0.148)	–	–	22.4
*Covariate relationships*					
Gestational age on F_AQ_ (%)	1.28 (25.0)	1.29 (0.65–1.92)	–	–	–
*Secondary parameters (median, 95%CI)*					
T_1/2 AQ_ (hours)	26.3 (24.7–28.6)				
AUC_AQ_ (h∙ng/mL)	283 (182–432)				
T_1/2 DEAQ_ (day)	14.1 (12.8–16.9)				
Day 7 _DEAQ_ (ng/mL)	63.3 (38.4–107)				
AUC _DEAQ_ (h∙μg/mL)	45.2 (27.5–78.3)				

^a^ %RSE was obtained from a bootstrap approach. Population estimates are given for a “typical” pregnant women weighting 70 kg with acute falciparum malaria. Ka is the absorption rate constant. MTT is the mean transit time. CL/F is the elimination clearance. V_C_/F is the central volume of distribution. Q/F is the inter‐compartment clearance. V_P_/F is the peripheral volume of distribution. F is the relative bioavailability. RUV is the residual error variance. T_1/2_ is the terminal elimination half‐life. AUC is the area under the concentration‐time curve from time zero to infinite. Day 7 DEAQ is the model‐predicted day 7 plasma concentration. Secondary‐parameter estimates were derived from the Empirical Bayes post‐hoc estimates. Coefficients of variation for inter‐individual variability (IIV) were calculated as 100 × (*e* ^variance^ − 1)^1/2^. Gestational age (GA) was implemented on relative oral bioavailability [1 + (*θ* × (GA‐24))]. 67 runs (out of 1000) with estimates near a boundary were omitted when calculating the bootstrap results.

**TABLE 3:** Final parameter estimates of piperaquine population pharmacokinetics in pregnant women.

Parameter	NONMEM Population estimates (%RSE^a^)	Bootstrap median (95%CI)	CV for IIV (%RSE^a^)	Bootstrap median (95%CI)	Shrinkage (%)
F (%)	100 fixed	–	33.8 (9.2)	33.6 (27.2–40.2)	41.0
MTT (h)	2.11 fixed	–	–	–	–
Number of transit compartments	2 fixed	–	–	–	–
CL/F (L/h)	69.9 (5.1)	69.3 (62.5–76.1)	–	–	–
V_C_/F (L)	4240 (36.0)	4010 (1600‐7600)	112 (29.3)	125 (37–308)	73.1
Q_1_/F (L/h)	265 (39.9)	243 (63–472)	–	–	–
V_p1_/F (L)	3880 (28.7)	3860 (1240–5730)	–	–	–
Q_2_/F (L/h)	103 (12.8)	100 (76–127)	–		–
V_p2_/F (L)	22,900 (7.9)	22,800 (19,900–26,700)	–	–	–
RUV	0.222 (9.5)	0.215 (0.177–0.256)	–		17.7
*Covariate relationships*					
Baseline parasites count on F (%)	−11.9 (19.3)	−12.1 (−16.2 to −7.3)	‐	–	–
Dose occasion on F (%)	23.7 fixed				
*Secondary parameters (median, 95%CI)*					
T_1/2_ (day)	17.0 (15.8–18.6)				
Day 7 (ng/mL)	39.0 (25.1–65.0)				
AUC (h∙μg/mL)	31.6 (20.6–50.0)				

^a^ %RSE was a obtained from a bootstrap approach. Population estimates are given for a “typical” pregnant women weighted 70 kg with acute falciparum malaria. MTT is the mean transit time. CL is the elimination clearance. V_C_ is the volume of distribution of the central compartment. V_P1_ and V_P2_ are the volume of distribution of the peripheral compartments. Q_1_ and Q_2_ are the inter‐compartment clearances. F is the relative oral bioavailability. T_1/2_ is the terminal elimination half‐life. Day 7 is the model‐predicted day 7 plasma concentration. AUC is the area under the concentration–time curve from time zero to infinity. Secondary‐parameter estimates were calculated from the Empirical Bayes post‐hoc estimates. Coefficients of variation for inter‐individual variability (IIV) were calculated as 100 × (*e* ^variance^–1)^1/2^. Relative standard errors (%RSE) were calculated as 100 × (standard deviation/mean). Baseline parasite counts (log scale) were implemented on relative oral bioavailability [1 + *θ* × (log(parasitemia)−2.83)]. Dose occasion was implemented on relative oral bioavailability [1 + *θ* × (OCC‐1)]. 36 runs (out of 1000) with estimates near a boundary were omitted when calculating the bootstrap results.

### Simulations

**FIGURE 3:**
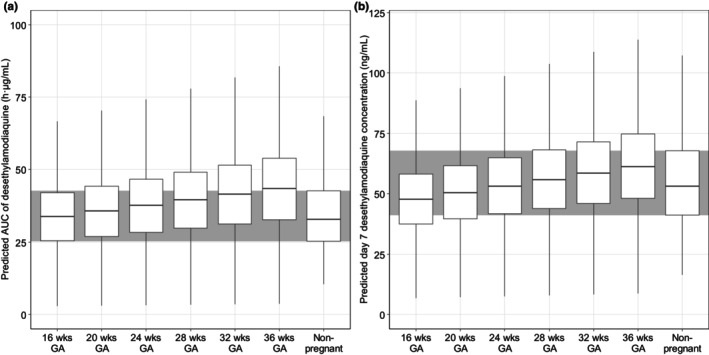
Predicted AUC (a) and day 7 concentration (b) of desethylamodiaquine according to different GA, based on 1000 hypothetical patients per 4 weeks of GA. The simulation is based on a typical pregnant woman with bodyweight of 70 kg. wks is weeks. AUC is the area under curve from zero to infinity.

The final population pharmacokinetic models were used for stochastic simulations (*n* = 1000 hypothetical patients for every 4 weeks of GA, all weighing 70 kg, with a baseline parasitemia of 676 parasites/μL) to evaluate the impact of pregnancy. Literature models in non‐pregnant women were used to simulate the exposure in this population. These simulations showed that the predicted AUC of desethylamodiaquine was 2.8%–32.2% higher in pregnant women at 16–36 weeks of pregnancy compared to that in non‐pregnant women, while day 7 concentration was comparable with a difference of −10.1% to 15.5% (Figure 3).

**FIGURE 4:**
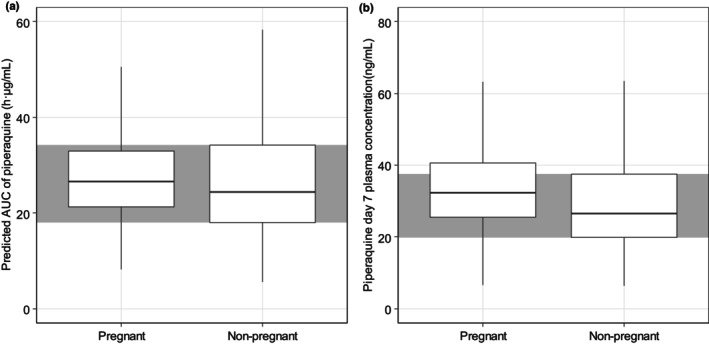
Predicted AUC (a) and Day 7 concentration (b) of piperaquine in pregnant and non‐pregnant women, based on 1000 hypothetical patients. The simulation is based on a typical pregnant woman with bodyweight of 70 kg and baseline parasitemia of 676/μL. AUC is the area under curve from zero to infinity.

As presented in Figure 4, the predicted AUC and day 7 concentrations of piperaquine were slightly higher in pregnant women compared to that in non‐pregnant women, with a difference of 10.0% and 20.7%, respectively.

## DISCUSSION

To our best knowledge, this is the largest population PK analysis of amodiaquine and piperaquine conducted in women in their second or third trimester of pregnancy with *P. falciparum* mono‐infection. In this study, we demonstrated that GA was significantly correlated to the absorption of amodiaquine and had no effects on the pharmacokinetic properties of piperaquine. Even with gestational age as a covariate on relative bioavailability, the exposure to the main active metabolite of amodiaquine (i.e., desethylamodiaquine) was overall comparable to that in non‐pregnant women.

The oral relative bioavailability is dependent on the gut bioavailability and the fraction of dose escaping hepatic extraction during the absorption into the systemic circulation. Several physiological factors can affect drug absorption, including gastric emptying, gut transit, uptake and efflux transporters, drug‐metabolizing enzymes, gastric pH, and porosity of tight junctions. Pregnancy could elevate gastric pH,^26^, ^30^ prolong small bowel transit time and therefore affect drug absorption.^7^, ^31^ Moreover, the activity of the hepatic and intestinal cytochrome P450 isoenzymes is reported to be increased during pregnancy, which could potentially impact drug metabolism and therefore the first‐pass drug metabolism.^32^ The increase in relative bioavailability of amodiaquine during pregnancy could be the result of one or many of these processes. In addition, amodiaquine mainly undergoes metabolism in the liver through CYP2C8 to its major metabolite, des‐ethylamodiaquine.^33^ Two previously published studies suggested pregnancy‐related induction of CYP2C8 protein concentrations.^34^, ^35^ The present study showed that the exposure to desethylamodiaquine was higher than that in non‐pregnant women, which could be attributed to elevated CYP2C8 enzyme levels during pregnancy. However, the quantified pregnancy effect was relatively small, with a maximum of 30% higher total exposure at a GA of 36 weeks compared to that in non‐pregnant women. This indicates a low risk of suboptimal concentrations due to pregnancy.

For piperaquine, CYP3A4 is the main metabolism enzyme^36^ and CYP3A expression is consistently and significantly increased (35%–38%) throughout pregnancy,^37^ theoretically resulting in lower drug exposure. However, a large pooled PK analysis of piperaquine based on 11 clinical studies (8776 samples from 728 individuals) showed CL was 55.4 L/h for a typical 70‐kg adult patient,^28^ which was close to our study (69.9 L/h). In this study, the predicted AUC and day 7 concentrations of piperaquine in pregnant women were comparable to that in non‐pregnant women. The present study also showed no significant change in PK exposure during the second and third trimester. A possible reason for such discrepancy between the theoretical assumption and our observation is not fully understood. However, the current study lacks data in the absorption phase, which makes it difficult to fully investigate the impact of pregnancy. Further studies are needed to address this question.

Malabsorption, due to impaired gastrointestinal function, has been demonstrated during the acute phase of a *P. falciparum* malaria infection. The pathophysiological mechanism behind this may partly be due to ischemic changes in the mucosa of the small bowel.^38^ Previous studies have shown increased relative bioavailability between each dosing occasion for both amodiaquine^9^ and piperaquine,^28^ which might be attributed to the recovery from malaria. The impact of different dose occasion was not investigated in our study, because most PK samples were collected after the last dose. In order to consider this effect, we fixed the dose occasion covariate to a literature value in the present model. In addition, we found that higher baseline parasitemia reduced the relative bioavailability in pregnant women, resulting in lower drug exposure in patients with a higher parasite burden and therefore more severe symptoms. This finding was consistent with a previous pooled population PK analysis of piperaquine, which indicated that relative bioavailability was correlated to disease severity.^28^

In the current study, the terminal half‐life of amodiaquine was longer than in previous reports (27.1 vs 13.7 h). This might be due to the sparse collection scheme in the current study, impacting the estimation of elimination clearance and volume of distribution. The half‐life of desethylamodiaquine was 13.7 days in the present study, which is in line with previous reports in pregnant women^9^ (13.7 vs 12.3 days) and postpartum women (12.6 days).^9^ The terminal half‐life of piperaquine was 19.6 days in this study, which is comparable to that observed in pregnant women in Sudan (*n* = 12, *t*_1/2_ = 23.4 days)^11^, Papua New Guinea (*n* = 32, *t*_1/2_ = 15.9 days^10^) and at the Thai‐Myanmar border (*n* = 24, *t*_1/2_ = 17.5 days)^13^. However, the half‐life reported in our study was longer than that reported in one study in pregnant women in Papua New Guinea (*n* = 30, 13.1 days^12^), which might be attributed to a discrepancy in sampling schemes.

This study has some key limitations. It did not include non‐pregnant women, and therefore the comparison between pregnant and non‐pregnant women had to be done using simulations from previously published large population PK meta‐models. Larger pharmacokinetic meta‐analyses, including all available pharmacokinetic data from pregnant and non‐pregnant women, should be encouraged to resolve these questions. Limited data were available in the absorption phase, which make it difficult to evaluate this phase of the drug concentration‐time profile.

## CONCLUSION

The total exposure to desethylamodiaquine and piperaquine in pregnant women was similar to that previously reported in non‐pregnant women, making dose adjustment unwarranted in women in their second or third trimester of pregnancy.

## AUTHOR CONTRIBUTIONS

J.D., R.M.H., and J.T. wrote the manuscript. H. Tagbor, H. Tinto, I.V., L.K.‐P., J.‐P.V., M.M., and U.D. designed the research. H. Tagbor, H. Tinto, I.V., V.M., L.K.‐P., J.‐P.V.G., M.N., M.M., S.H., R.R., and U.D. performed the research. J.D., R.M.H., and J.T. analyzed the data. All authors reviewed the final manuscript.

## FUNDING INFORMATION

This work was supported by the European and Developing Countries Clinical Trials Partnership [IP.2007.31080.001], the Malaria in Pregnancy Consortium (which is funded through a grant from the Bill and Melinda Gates Foundation to the Liverpool School of Tropical Medicine). This work was also partly supported by the Wellcome Trust [220211]. The funders had no part in the study design, implementation and analysis of the result or the decision to publish this manuscript. For the purpose of open access, the author has applied a CC BY public copyright license to any Author Accepted Manuscript version arising from this submission.

## CONFLICT OF INTEREST STATEMENT

All authors declared no competing interests for this work.

## Supporting information


Data S1.
