# Supplementary material for: Population pharmacokinetics of amodiaquine and piperaquine in African pregnant women with uncomplicated Plasmodium falciparum infections
Source: CPT Pharmacometrics Syst Pharmacol. 2024 Sep 3;13(11):1893–903. doi: 10.1002/psp4.13211 (PMC11578137; doi:10.1002/psp4.13211)
Supplement: Supplementary file 1 — Data S1. [file PSP4-13-1893-s001.docx]

**Supplementary Material**

**Population pharmacokinetics of amodiaquine and piperaquine**

**in African pregnant women with uncomplicated Plasmodium falciparum infections**

*Junjie Ding, Richard M. Hoglund, Harry Tagbor, Halidou Tinto, Innocent Valéa, Victor Mwapasa, Linda Kalilani-Phiri, Jean-Pierre Van Geertruyden, Michael Nambozi, Modest Mulenga, Sebastian Hachizovu, Raffaella Ravinetto, Umberto D’Alessandro, Joel Tarning*

**Figure S1**. Graphical overview of the structural population pharmacokinetic model for amodiaquine and desethylamodiaquine.

**Figure S2**. Graphical overview of the structural population pharmacokinetic model for piperaquine.

**Figure S3.** Goodness-of-fit plots for the final population pharmacokinetic model describing amodiaquine (A, B, C and D) and desethylamodiaquine (E, F, G and H).

**Figure S4**. Goodness-of-fit plots for the final population pharmacokinetic model describing piperaquine.

**Figure S5**. Prediction-corrected visual predictive check of the final population pharmacokinetic model for amodiaquine (A) and desethylamodiaquine (B) across gestational ages.

**Figure S6**. Prediction-corrected visual predictive check of the final population pharmacokinetic model for piperaquine across gestational ages.

**NONMEM Code**

**Appendix 1.** NONMEM code for the final population PK model of amodiaquine and desethylamodiaquine

**Appendix 2.** NONMEM code for the final population PK model of piperaquine

**
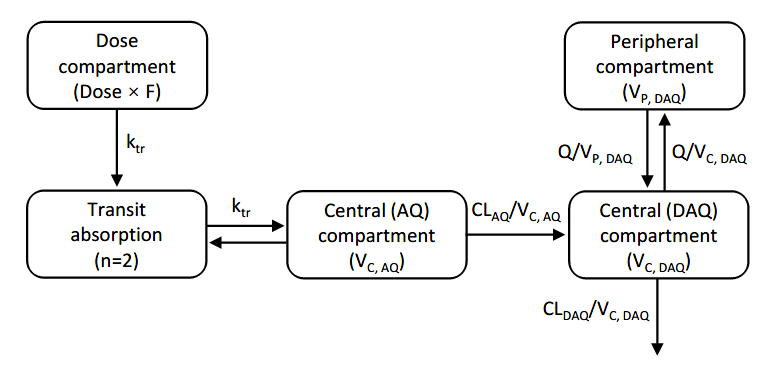
**

**Figure S1. Graphical overview of the structural population pharmacokinetic model for amodiaquine and desethylamodiaquine.**

AQ: amodiaquine, DAQ: desethylamodiaquine. K_a_ is the first order absorption rate constant. K_tr_ is the rate constant between transit compartments, defined as KTR = (n+1)/MTT. CL is the elimination clearance. V_C_ is the volume of distribution of the central compartment. V_P_ is the volume of distribution of the peripheral compartment. Q is the inter-compartment clearance. F is the relative oral bioavailability.

**
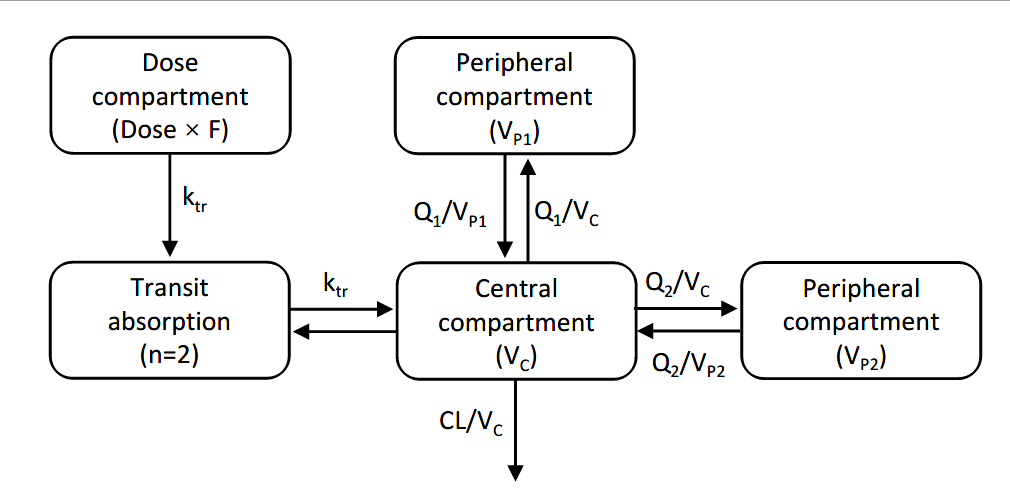
**

**Figure S2. Graphical overview of the structural population pharmacokinetic model for piperaquine.**

k_TR_ is the rate constant between transit compartments, defined as KTR = (n+1)/MTT. CL is the elimination clearance. V_C_ is the volume of distribution of the central compartment. V_P1_ and V_P2_ are the volume of distribution of the first and second peripheral compartment. Q_1_ and Q_2_ are the inter-compartment clearance for the first and second peripheral compartment. F is the relative oral bioavailability.

**Figure S3. Goodness-of-fit plots for the final population pharmacokinetic model describing amodiaquine (A, B, C and D) and desethylamodiaquine (E, F, G and H).**

A: Observed plasma concentrations versus population predicted concentrations (A, E) and against individually predicted concentrations (B, F). Conditionally weighted residuals plotted versus population predicted concentrations (C, F) and time (D, H). The solid lines are the line of identity, and the dashed lines are the locally weighted least squares regression line.

**Figure S4. Goodness-of-fit plots for the final population pharmacokinetic model describing piperaquine.**

A: Observed plasma concentrations versus population predicted concentrations (A) and against individually predicted concentrations (B). Conditionally weighted residuals plotted versus population predicted concentrations (C) and time (D). The solid lines are the line of identity, and the dashed lines are the locally weighted least squares regression line

**Figure S5. Prediction-corrected visual predictive check of the final population pharmacokinetic model for amodiaquine (A) and desethylamodiaquine (B) across gestational ages.**

Open circles represent the observations, and solid lines represent the 5th, 50th, and 95th percentiles of the observed data. The shaded areas represent the 95% confidence intervals around the simulated 5th, 50th, and 95th percentiles.

**Figure S6. Prediction-corrected visual predictive check of the final population pharmacokinetic model for piperaquine across gestational ages.**

Open circles represent the observations, and solid lines represent the 5th, 50th, and 95th percentiles of the observed data. The shaded areas represent the 95% confidence intervals around the simulated 5th, 50th, and 95th percentiles.

**Appendix 1.**

**NONMEM code for the final population PK model of amodiaquine and desethylamodiaquine**

$INPUT

ID TIME OCC DV CMT AMT MDV EVID AGE GA PARA PA GAME WT

$DATA

NM_AQ.csv

$SUBROUTINE

ADVAN5 TRANS1

$MODEL

COMP = (1) ; Dose

COMP = (2) ; AQ central compartment

COMP = (3) ; DAQ central compartment

COMP = (4) ; DAQ peripheral compartment

COMP = (5) ; Transit compartment 1

COMP = (6) ; Transit compartment 2

$PK

CGA = (1 + THETA (10)*(GA - 24)) ; Linear covariate relationship for gestational age

TVCL = THETA (1) * ((WT/70) ** 0.75) ; Population AQ clearance

CL = TVCL * EXP(ETA (1)) ; Individual AQ clearance

TVV2 = THETA (2) * (WT/70) ; Population AQ central volume

V2 = TVV2 * EXP(ETA (2)) ; Individual AQ central volume

TVKA = THETA (3) ; Population absorption rate

KA = TVKA * EXP(ETA (3)) ; Individual absorption rate

TVMTT = THETA (4) ; Population mean transit time

MTT = TVMTT * EXP(ETA (4)) ; Individual mean transit time

TVCL2 = THETA (5) * ((WT/70) ** 0.75) ; Population DAQ clearance

CL2 = TVCL2 * EXP(ETA (5)) ; Individual DAQ clearance

TVV3 = THETA (6) * (WT/70) ; Population DAQ central volume

V3 = TVV3 * EXP(ETA (6)) ; Individual DAQ central volume

TVQ = THETA (7) * ((WT/70) ** 0.75) ; Population DAQ inter-compartment clearance

Q = TVQ * EXP(ETA (7)) ; Individual DAQ inter-compartment clearance

TVV4 = THETA (8) * (WT/70) ; Population DAQ peripheral volume

V4 = TVV4 * EXP(ETA (8)) ; Individual DAQ peripheral volume

TVF1= THETA (9) * CGA ; Population relative bioavailability

F1 = TVF1 * EXP(ETA (9)) ; Individual relative bioavailability

NN = 2 ; Number of transit compartments

KTR = (NN+1)/MTT ; Transit rate constant

K15 = KTR ; Transit rate constant (COMP 1 --> 5)

K56 = KTR ; Transit rate constant (COMP 5 --> 6)

K62 = KA ; Absorption rate constant (COMP 6 --> 2)

K23 = CL/V2 ; AQ elimination rate constant (COMP 2 --> 3)

K30 = CL2/V3 ; DAQ elimination rate constant (COMP 3 --> 0)

K34 = Q/V3 ; DAQ distribution rate constant (COMP 3 --> 4)

K43 = Q/V4 ; DAQ distribution rate constant (COMP 4 --> 3)

$ERROR

IF (CMT.EQ.2) W=SQRT(SIGMA (1,1)) ; AQ residual error

IF (CMT.EQ.3) W=SQRT(SIGMA(2,2)) ; DAQ residual error

IF (F.GT.0) IPRED = LOG(F) ; Natural logarithm of predictions

IRES = IPRED - DV ; Individual residual error

IWRES = IRES/W ; Individually weighted residual error

IF (CMT.EQ.2) Y = IPRED + EPS(1) ; AQ additive residual error

IF (CMT.EQ.3) Y = IPRED + EPS(2) ; DAQ additive residual error

$THETA

(0, 6180) ; 1. AQ Clearance

(0, 269000) ; 2. AQ central volume

(0.589) FIX ; 3. Absorption rate

(0.236) FIX ; 4. Mean transit time

(0, 37.9) ; 5. DAQ clearance

(0, 872) ; 6. DAQ central volume

(0, 84.1) ; 7. DAQ inter-compartment clearance

(0, 13500) ; 8. DAQ peripheral volume

(1) FIX ; 9. Relative bioavailability

(0, 0.0125) ; 10. Gestational age on relative bioavailability

$OMEGA

0 FIX ; 1. IIV AQ Clearance

0 FIX ; 2. IIV AQ central volume

0 FIX ; 3. IIV absorption rate

0 FIX ; 4. IIV mean transit time

0.0315 ; 5. IIV DAQ clearance

1.61 ; 6. IIV DAQ central volume

0 FIX ; 7. IIV DAQ inter-compartment clearance

0 FIX ; 8. IIV DAQ peripheral volume

0.0905 ; 9. IIV Relative bioavailability

$SIGMA

0.265 ; 1. AQ additive residual error

0.127 ; 2. DAQ additive residual error

$ESTIMATE

MAXEVAL=9990 METHOD=COND INTE NOABORT PRINT=1 POSTHOC

**Appendix 2.**

**NONMEM code for the final population PK model of piperaquine**

$INPUT

ID TIME OCC DV CMT AMT MDV EVID AGE GA PARA PA GAME WT

$DATA

NM_PQ.csv

$SUBROUTINE

ADVAN5 TRANS1

$MODEL

COMP = (1) ; Dose

COMP = (2) ; Central compartment

COMP = (3) ; First peripheral compartment

COMP = (4) ; Second peripheral compartment

COMP = (5) ; Transit compartment 1

COMP = (6) ; Transit compartment 2

$PK

F1PA = (1 + THETA (9) * (PA - 2.83)) ; Linear covariate relationship for parasitemia

F1COVD = (1 + THETA (10) * (OCC - 1)) ; Linear covariate relationship for dose occasion

TVCL = THETA (1) * ((WT/70) ** 0.75) ; Population clearance

CL = TVCL * EXP(ETA (1)) ; Individual clearance

TVV2 = THETA (2) * (WT/70) ; Population central volume

V2 = TVV2 * EXP(ETA (2)) ; Individual central volume

TVMTT = THETA (3) ; Population mean transit time

MTT = TVMTT * EXP(ETA (3)) ; Individual mean transit time

TVQ = THETA (4) * ((WT/70) ** 0.75) ; Population inter-compartment clearance 1

Q = TVQ * EXP(ETA (4)) ; Individual inter-compartment clearance 1

TVV3 = THETA (5) * (WT/70) ; Population peripheral volume 1

V3 = TVV3 * EXP(ETA (5)) ; Individual peripheral volume 1

TVQ2 = THETA (6) * ((WT/70) ** 0.75) ; Population inter-compartment clearance 2

Q2 = TVQ2 * EXP(ETA (6)) ; Population inter-compartment clearance 2

TVV4 = THETA (7) * (WT/70) ; Population peripheral volume 2

V4 = TVV4 * EXP(ETA (7)) ; Individual peripheral volume 2

TVF1= THETA (8) * F1PA *F1COVD ; Population relative bioavailability

F1 = TVF1 * EXP(ETA (8)) ; Individual relative bioavailability

NN = 2 ; Number of transit compartments

KTR = (NN+1)/MTT ; Transit rate constant

K15 = KTR ; Transit rate constant (COMP 1 --> 5)

K56 = KTR ; Transit rate constant (COMP 5 --> 6)

K62 = KTR ; Transit rate constant (COMP 6 --> 2)

K20 = CL/V2 ; Elimination rate constant (COMP 2 --> 0)

K23 = Q/V2 ; Distribution rate constant (COMP 2 --> 3)

K32 = Q/V3 ; Distribution rate constant (COMP 3 --> 2)

K24 = Q2/V2 ; Distribution rate constant (COMP 2 --> 4)

K42 = Q2/V4 ; Distribution rate constant (COMP 4 --> 2)

$ERROR

W=SQRT(SIGMA(1,1)) ; Residual error

IF (F.GT.0) IPRED = LOG(F) ; Natural logarithm of predictions

IRES = IPRED-DV ; Individual residual error

IWRES = IRES/W ; Individually weighted residual error

Y = IPRED + EPS(1) ; Additive residual error

$THETA

(0, 55.7) ; 1. Clearance

(0, 3240) ; 2. Central volume

(2.11) FIX ; 3. Mean transit time

(0, 189) ; 4. Inter-compartment clearance 1

(0, 3120) ; 5. Peripheral volume 1

(0, 78.2) ; 6. Inter-compartment clearance 2

(0, 18100) ; 7. Peripheral volume 2

(1) FIX ; 8. Relative bioavailability

(-0.2, -0.119) ; 9. Parasitemia on relative bioavailability

(0.237) FIX ; 10. Dose occasion on relative bioavailability

$OMEGA

0 FIX ; 1. IIV clearance

0.833 ; 2. IIV central volume

0 FIX ; 3. IIV mean transit time

0 FIX ; 4. IIV inter-compartment clearance 1

0 FIX ; 5. IIV peripheral volume 1

0 FIX ; 6. IIV inter-compartment clearance 2

0 FIX ; 7. IIV peripheral volume 2

0.106 ; 8. IIV relative bioavailability

$SIGMA

0.219 ; 9. Additive residual error

$ESTIMATE

MAXEVAL=9990 METHOD=COND INTE NOABORT PRINT=1 POSTHOC
